# A Spatial Cluster Analysis of Tractor Overturns in Kentucky from 1960 to 2002

**DOI:** 10.1371/journal.pone.0030532

**Published:** 2012-01-24

**Authors:** Daniel M. Saman, Henry P. Cole, Agricola Odoi, Melvin L. Myers, Daniel I. Carey, Susan C. Westneat

**Affiliations:** 1 Department of Epidemiology, College of Public Health, University of Kentucky, Lexington, Kentucky, United States of America; 2 Department of Preventive Medicine and Environmental Health, Southeast Center for Agricultural Health and Injury Prevention, College of Public Health, University of Kentucky, Lexington, Kentucky, United States of America; 3 Department of Biomedical & Diagnostic Sciences, College of Veterinary Medicine, University of Tennessee, Knoxville, Tennessee, United States of America; 4 Department of Kentucky Geological Survey, University of Kentucky, Lexington, Kentucky, United States of America; University of Swansea, United Kingdom

## Abstract

**Background:**

Agricultural tractor overturns without rollover protective structures are the leading cause of farm fatalities in the United States. To our knowledge, no studies have incorporated the spatial scan statistic in identifying high-risk areas for tractor overturns. The aim of this study was to determine whether tractor overturns cluster in certain parts of Kentucky and identify factors associated with tractor overturns.

**Methods:**

A spatial statistical analysis using Kulldorff's spatial scan statistic was performed to identify county clusters at greatest risk for tractor overturns. A regression analysis was then performed to identify factors associated with tractor overturns.

**Results:**

The spatial analysis revealed a cluster of higher than expected tractor overturns in four counties in northern Kentucky (RR = 2.55) and 10 counties in eastern Kentucky (RR = 1.97). Higher rates of tractor overturns were associated with steeper average percent slope of pasture land by county (p = 0.0002) and a greater percent of total tractors with less than 40 horsepower by county (p<0.0001).

**Conclusions:**

This study reveals that geographic hotspots of tractor overturns exist in Kentucky and identifies factors associated with overturns. This study provides policymakers a guide to targeted county-level interventions (e.g., roll-over protective structures promotion interventions) with the intention of reducing tractor overturns in the highest risk counties in Kentucky.

## Introduction

The earliest accounts of overturns of gasoline-powered tractors occurred when 1914 vintage Little Bull tractors overturned as they were turned uphill and to the right [1], [2]. This tricycle tractor had a large, heavy rear drive wheel on the right side that resulted in an offset center-of-gravity, giving it this propensity. Terrain was also a principle factor in these overturns. Overturns have long been associated with slopes, ditches, or bad ground conditions that precipitate tractor instability [3]. Indeed, when a tractor moves from a level position onto slopes, instability becomes a hazard [4], but more broadly, topography is a recognized risk factor associated with tractor overturns including obstacles, obstructions, slippery surfaces, hills and slopes, ditches, and embankments [5].

Agricultural tractor overturns without rollover protective structures (ROPS) are the leading cause of farm fatalities in the United States [6]. While tractor overturn fatalities have been associated with the decedent's age, type of farm, region of the country, the victim's relationship to the farm, and the gender of the tractor operator [7], [8], rarely have investigations addressed the physical features of the tracts of land, i.e., terrain. The imperative for this study originated when an analysis of Census of Fatal Occupational Injuries (CFOI) data identified six states (Illinois, Kentucky, Ohio, Pennsylvania, Tennessee, and West Virginia) within or near the Appalachian mountain range that had the highest rates of agricultural tractor overturn deaths within the United States [9]. This suggested that topographic features, along with smaller farms, were associated with tractor overturns [10].

Terrain and tractor characteristics as risk factors associated with tractor overturns have been anecdotally studied, but in contrast, our current study examines the issue using novel spatial statistical techniques incorporating geographic information systems (GIS), Kulldorff's spatial scan statistic [11], and traditional regression modeling. As an extension of the Cole et al (2009) paper that provided descriptive statistics on the six states with the highest rates of overturn deaths, this study examines whether overturns are randomly distributed or are clustered in certain regions of Kentucky [10]. It is hypothesized that tractor overturns are not spatially randomly distributed in Kentucky, and are associated with physiographic terrain characteristics such as steeper slopes.

Though Kulldorff's spatial scan statistic has only recently been adopted by public health researchers across several fields [12]–[15], to our knowledge, no studies have incorporated the spatial scan statistic in identifying high-risk areas for tractor overturns. Thus, the purpose of this ecological study was to identify geographic areas at the highest risk for tractor overturns and to identify factors, including physiographic and tractor characteristics, associated with overturns at the county level. This study provides an analytical framework using spatial statistics and regression modeling to identify and target areas with the highest risk for overturns. Moreover, knowing the counties at greatest risk for overturns can guide resources and interventions to those counties. Other high-risk states might also apply these same cluster analysis techniques for future state-to-state comparison.

## Methods

### Data sources

This research was approved by the University of Kentucky Institutional Review Board (IRB #: 01-0710-P2B).

Three sources of data were used for this study. The first source is the Kentucky Farm Tractor Overturn Telephone Survey (KY T/O Survey). This survey provided estimates of total farm tractor overturns and the frequency of six classes of overturn injury outcomes for tractors with and without rollover protective structures (ROPS). The sample was an 8% population-based random sample, with sampling frame, design, and telephone survey completed by the Kentucky Agricultural Statistics Service (KASS). KASS constructed the sampling frame using its 2001 updated 1997 Census of Agriculture full list of farms in Kentucky. Kentucky farms were randomly sampled proportional to their number in each of the six agricultural districts. The KY T/O Survey was administered during October 2002. The KASS statisticians randomly sampled farms across Kentucky's six agricultural districts stratified by farm size and annual value of sales. Then, KASS enumerators began calling farms in random order. The survey was stopped when 8% (6,063) of Kentucky farmers completed the survey. The 6,063 completed surveys by Kentucky farmers represent a 79% response rate. The 40-item telephone survey collected information at the county level from Kentucky farmers about each farm's history of overturns. A total of 551 (9.1%) farms reported overturns, whereas 5,512 (90.9%) farms reported no overturn events in the history of their farm. The period from 1925 to 1959 accounted for only 47 (7.8%) of the total 603 overturns, while 556 (92.2%) overturns occurred from 1960 to 2002. Thus, all analyses used data from the 1960 to 2002 period [16]. Informed consent was obtained over the telephone and documented on paper. The survey interview protocol included as the first topic addressed an informed consent statement that advised those who were contacted (a) the purpose of the survey (b) a request to speak to the farm primary operator or other person who was responsible for farm operation, (c) the sample characteristics (KY farmer principal farm operators as listed in the 2001–2002 USDA KY farm Census) (d) the number of farms to be surveyed (6,000 or 8% of KY farms) and that (e) the farmers called were randomly sampled, (f) that the survey items addressed the number of tractors on their farms, (g) the number of tractor overturns in the history of their farm, (h) questions about the type of overturn, (i) description of the type and extent of any injuries to the operator the approximate length of the survey and the time required to complete it, (j) a statement that the person interviewed was free to skip any questions he or she did not want to answer, and (k) could end the interview at any point during the survey. The University of Kentucky Institutional Review Board approved this consent procedure.

The second data source is the 2007 Census of Agriculture. For each Kentucky county, we abstracted the total number of tractors, the number of tractors with less than 40 horsepower, the number of tractors with 100 horsepower or more [17], and the median size of farms in acres [18]. The total number of tractors by county was used in the spatial cluster investigation to provide denominator data, and in the regression model to provide rates by county. The number of tractors with less than 40 horsepower, 100 horsepower or more, and median size of farms were used in the regression model.

Finally, to account for Kentucky's diverse physiographic characteristics, the Kentucky Geological Survey provided county-level data on average percent slope of pasture land, percent crop and pasture land, and stream miles per square mile of crop and pasture land. Data for these three physiographic variables were derived from the National Hydrography Dataset, NHD24 [19], 10-meter digital elevation data [20], and Kentucky 2005 Land Cover – Anderson Level II data [21]. We used county data from the KYGEONET GIS database [22], extracted cultivated crops and pasture/hay data from the land cover data and calculated by county crop and pasture land percentages. To get a measure of “dissected drainage”, we intersected the NHD24 stream data with the crop and pasture land to get miles of streams per square mile of crop and pasture land. We calculated the percent land slope on a 10-meter grid using the Digital Elevation Model data, intersected this with counties, calculated average county slope, and then intersected pasture and slope data and calculated average pasture slope by county. We used ArcGIS v10 (ESRI, Inc, Redlands, CA.) to derive these three physiographic county-level variables [23].

### Geographic analysis

Smoothed cumulative incidence tractor overturn rates were mapped by county aggregations (n = 120). The heterogeneity of variances of overturn rates were adjusted using Spatial Empirical Bayesian (SEB) rate smoothing [24]. Given that tractor population sizes vary considerably by county, overturn rates from counties of low population have greater variance than counties with higher populations [25]. SEB smoothing adjusts for the high variances of areas with low population [26] by adjusting the rates from low population areas toward a local mean based on a spatial weights matrix [25]–[27]. This technique was implemented in GeoDa v0.95i [28] using a first-order queen contiguity spatial weights matrix [29].

The geographic boundary files used in this study were downloaded from the United States Census, TIGER, Geodatabase [30]. ArcGIS v10 [23] was used to create the cartographic displays, with single hue color schemes generated by ColorBrewer.org [31], and with grouping of data into classes/categories based on natural breaks in the data using the Jenks natural breaks optimization algorithm [32].

### Spatial scan statistic cluster detection

The detection of high-risk local spatial clusters of tractor overturns was performed using Kulldorff's 2-dimensional spatial scan statistic [11], and implemented in SaTScan v9.1 [33]. The advantages of using the scan statistic over simple comparisons of standardized incidence rates within clusters versus outside clusters include controlling for covariates, adjusting for multiple comparisons and various population sizes, and limiting preselection bias by not specifying *a priori* the observed set of cases within a cluster [34], [35]. The spatial scan statistic detects clusters by using circular windows with continuously varying radii from zero to a user-specified maximum that move across the geographic centroids (grid reference) of counties in the study area comparing the number of cases within each circular window to the number of expected cases assuming that cases were completely spatially randomly distributed across the study area [35], [36]. Significance testing is performed using Monte Carlo simulation where the null hypothesis of no cluster is rejected at α level of 0.05 if the simulated p-value is less than or equal to 0.05 [35]. A significant high-risk cluster is interpreted as having an increased risk of tractor overturns within the circular window relative to outside [37].

For the purely spatial cluster analysis, a discrete Poisson probability model was used to scan for non-overlapping geographical areas (counties) with statistically significant high rates of overturns. Given that the results of this analysis are sensitive to model parameters, and the goal was to identify small county clusters of greatest risk, similar to another study [38], a maximum spatial cluster size of 10% of the total population at risk was used. A maximum spatial cluster size of 10% indicates that the circular window will expand from zero up to a maximum of 10% of the total population at risk across the study period scanning for clusters, of which the most likely is that cluster that maximizes the log likelihood ratio. Cartographic displays of spatial clusters were made using ArcGIS v10 [23].

### Multiple linear regression modeling of tractor overturn rates

This is an ecological study with the unit of analysis being Kentucky counties; thus, the results can be interpreted only at the county level and not at the individual farm level. The outcome of interest in this modeling was continuous, reflecting tractor overturns per 100,000 tractors by county. Univariate associations of continuous variables with the outcome were assessed using Pearson's correlation coefficients and regression modeling of each variable one at a time against the outcome. Only variables with significant associations based on a moderate p-value (p<0.20) were considered in the multivariable modeling process. The assumptions of linearity of continuous variables with the outcome, constant variance of the errors, normality of the errors, and a mean of zero of the errors were checked using graphical methods. All the variables met the assumptions. In the final model, we used a Moran's I test utilizing a first-order queen contiguity spatial weights matrix in GeoDa v0.95i to test whether the outcome was spatially independent (i.e., observations are no longer spatially correlated after controlling for variables). A non-significant Moran's I value meant that the assumption of spatially independent observations of the outcome was met.

The modeling process started with a full model including all six variables as they were all independently associated with the outcome at the significance level of p<0.20. Next, we removed the variable with the highest non-significant p-value (significance set at p<0.05). This was continued until the final model contained only significant variables. Also, variables were assessed for confounding if their removal resulted in a greater than 20% change in the remaining variables in the model. Two-way interactions were not tested during the model building process. However, two-way interaction terms were assessed for significance in the final main-effects model. We assessed goodness of fit of the model using *R*-square and adjusted *R*-square. Finally, though Kentucky has 120 counties, the model includes only 119 counties as one county, Martin, was considered an extreme outlier. However, all counties were included in the cluster analysis as the results are not as sensitive to outliers as linear regression. Regression analyses were performed in SAS v9.3 (SAS Institute Inc, Cary, NC) [39].

## Results

### Description of tractor overturns

**Figure 1:**
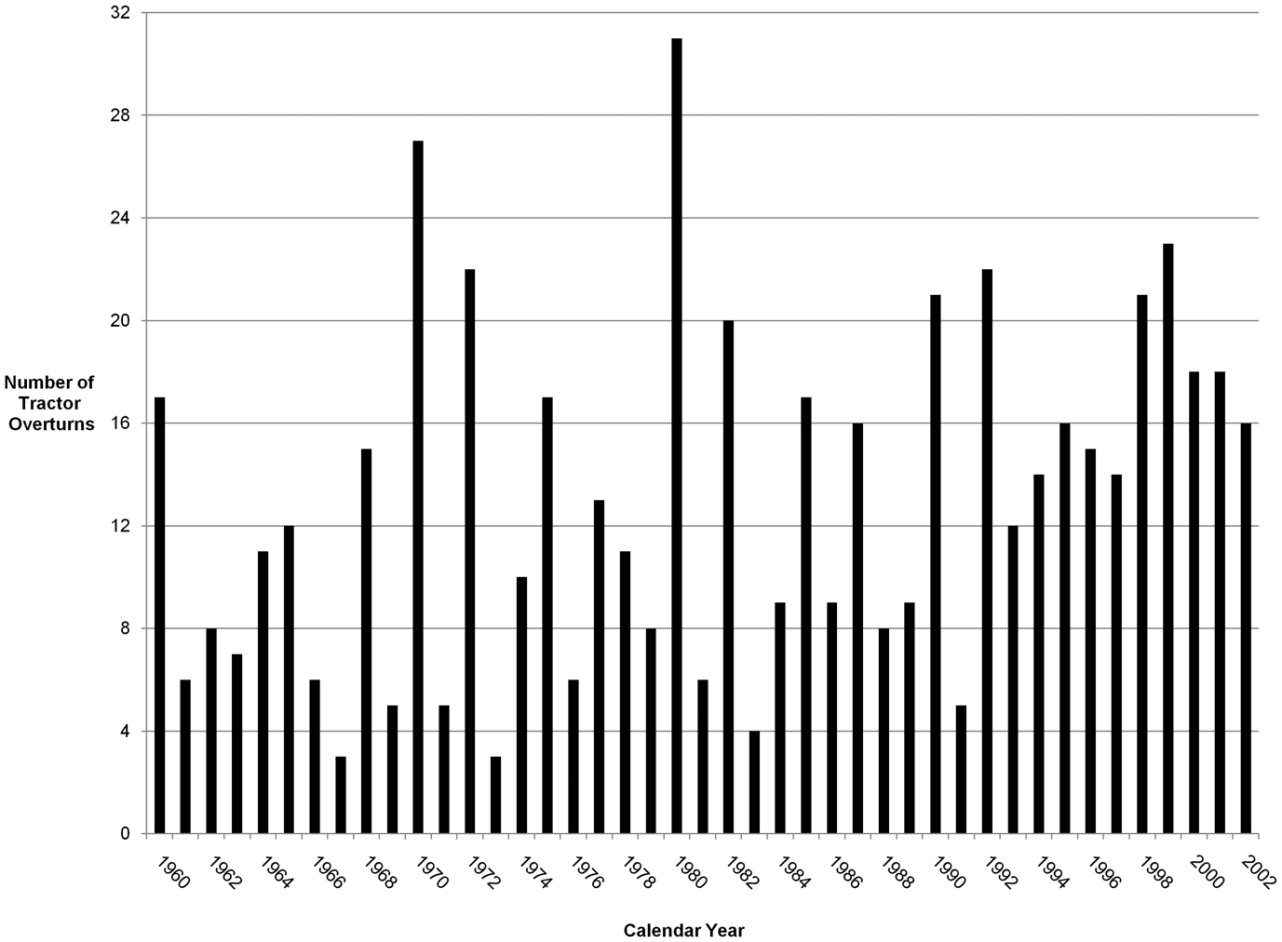
Tractor overturns by year in Kentucky, 1960–2002. Source: Kentucky Tractor Overturn Survey (KY T/O) (15).

Based on the 8% sample of Kentucky farms taken from the KY T/O Survey, there were 556 tractor overturns from 1960 to 2002. The annual tractor overturn rate was 8.0 per 100,000 tractors. Figure 1 shows the distribution of overturns by year, with several peaks in 1970 (27 overturns, 16.7 per 100,000 tractors), 1980 (31overturns, 19.1 per 100,000 tractors), and 1999 (23 overturns, 14.2 per 100,000 tractors).

### Spatial distribution of tractor overturns

**Figure 2:**
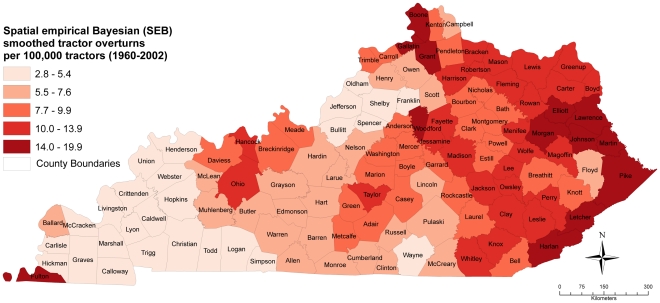
Spatial Empirical Bayes' (SEB) smoothed tractor overturn rates in Kentucky, 1960–2002.

The median SEB smoothed rate was 8.3 overturns per 100,000 tractors (range: 3–20). The counties with the highest SEB tractor overturn rates (greater than 16.3 overturns per 100,000 tractors) included Elliott (16.4/100,000), Fulton (16.4/100,000), Grant (16.6/100,000), Martin (17.6/100,000), Boone (19.1/100,000), and Pike (19.9/100,000) (Figure 2).

### Spatial clusters of high risk tractor overturns

**Figure 3:**
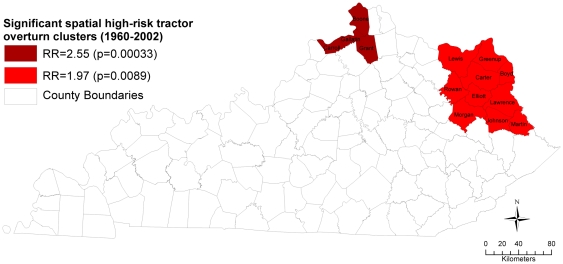
Significant spatial high-risk tractor overturn clusters in Kentucky, 1960–2002. *Note:* RR = relative risk; Interpreted as increased risk inside relative to outside the cluster.

Table 1 displays results of the spatial cluster analysis. Two significant (p<0.01) high-risk spatial clusters were identified. The most likely high-risk spatial cluster was comprised of four counties (Boone, Carroll, Gallatin, and Grant) in northern Kentucky (Figure 3). There were 38 overturns that occurred in this spatial cluster, while there were an expected 15.53 overturns. The tractor population within the most likely cluster is at a 2.55 times greater risk (relative risk, RR = 2.55; p = 0.00033) of overturns relative to the tractor population outside the cluster. Additionally, the counties within the most likely high-risk spatial cluster had an estimated 19.8 overturns per 100,000 tractors annually (Table 1). A secondary high-risk spatial cluster was found among ten counties (Boyd, Carter, Elliott, Greenup, Johnson, Lawrence, Lewis, Martin, Morgan, Rowan) in eastern Kentucky (Figure 3). This cluster had 49 overturns with 25.95 expected overturns, and a 97% increased risk (RR = 1.97, p = 0.00890) of overturns than outside the cluster. This secondary high-risk cluster had an estimated 15.1 overturns per 100,000 tractors annually (Table 1).

**Table 1:** Purely spatial high-risk clusters of tractor overturns in Kentucky, 1960–2002.

Type of cluster	Counties	Observed cases	Expected cases	Tractor Population	Annual cases/100,000 Tractors	Relative risk (RR)	*p*-Value	Log likelihood ratio
Most likely	Boone, Carroll, Gallatin, Grant	38	15.53	4531	19.5	2.55	0.00033	12.00
Secondary	Boyd, Carter, Elliott, Greenup, Johnson, Lawrence, Lewis, Martin, Morgan, Rowan	49	25.95	7569	15.1	1.97	0.00890	8.61

*Note:* The purely spatial analysis used a 10% spatial window and 19,999 Monte Carlo replications.

All 120 counties are included in the analysis.

The scan statistic scanned only for high-risk areas, and only significant clusters were reported.

Table 2 compares the most likely and secondary spatial clusters to the rest of Kentucky. No statistically significant differences in year of overturns were observed for inside versus outside the clusters. The cumulative incidence rate inside the most likely high-risk spatial cluster was 17.4 overturns per 100,000 tractors (range 11.3–24.1), while outside the cluster the rate was 9.1 overturns per 100,000 tractors (range 0–101.1). The cumulative incidence rate inside the secondary high-risk spatial cluster was 22.5 overturns per 100,000 tractors (range 0–101.1), while outside the cluster the rate was 8.1 overturns per 100,000 tractors (range 0–31.9).

**Table 2:** Descriptive characteristics of tractor overturns inside and outside purely spatial high-risk clusters.

	Within Most Likely High-Risk Spatial Cluster *(column % of tractor overturns within cluster)*	Excluding counties in cluster *(column % of tractor overturns outside cluster)*	Pearson χ2 (df)	Within Secondary High-Risk Spatial Cluster *(column % of tractor overturns within cluster)*	Excluding counties in cluster *(column % of tractor overturns outside cluster)*	Pearson χ2 (df)
*Counties*	Boone, Carroll, Gallatin, Grant	-		Boyd, Carter, Elliott, Greenup, Johnson, Lawrence, Lewis, Martin, Morgan, Rowan	-	
*Year*						
1960–1969	8 (21)	82 (16)	6.504, p = 0.165 (4)	7 (14)	83 (16)	2.751, p = 0.600 (4)
1970–1979	12 (32)	110 (21)		15 (31)	107 (21)	
1980–1989	3 (8)	126 (24)		10 (20)	119 (23)	
1990–1999	11 (29)	152 (29)		14 (29)	149 (29)	
2000–2002	4 (10)	48 (9)		3 (6)	49 (10)	
Total Tractor Overturns	38 (100)	518 (100)		49 (100)	507 (100)	
*Cumulative incidence rates per 100,000 persons*						
Average (SD)	17.4 (5.6)	9.1 (10.6)		22.5 (28.1)	8.1 (6.2)	
Median	18.2	7.4		16.2	7.2	
Range	11.3–24.1	0–101.1		0–101.1	0–31.9	

### Predictors of tractor overturns

**Figure 4:**
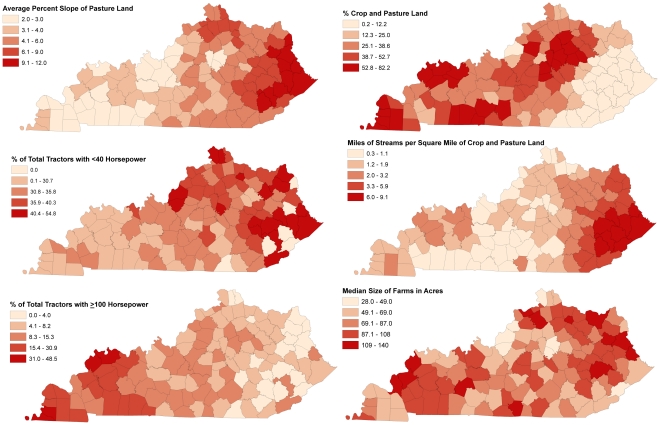
County level tractor characteristics and physiographic characteristics in Kentucky.

Table 3 presents descriptive statistics of both the dependent and independent variables. Six county-level physiographic and tractor characteristic variables were assessed in the model. Figure 4 shows the spatial distribution of the independent variables in Table 3, with apparent trends of higher values of average slope of pasture land, percent of total tractors with less than 40 horsepower, and miles of streams per square mile of crop and pasture land in eastern Kentucky. Conversely, percent crop and pasture land and the percent of total tractors with 100 horsepower or more have noticeably higher values in western than eastern Kentucky.

**Table 3:** Descriptive statistics of county level estimates of tractor overturn rates, tractor characteristics, and physiographic characteristics in Kentucky.

*n = 119 counties*				
	Mean (SD)	Median	Q1 - 25%	Q3 - 75%
*Dependent variable*				
Tractor overturns per 100,000 tractors	8.5 (6.4)	7.4	4.1	11.7
*Independent variables*				
Average percent slope of pasture land by county	5.3 (2.3)	5.0	4.0	6.0
Percent of total tractors with <40 horsepower by county	32.9 (9.7)	34.3	30.0	37.8
Percent of total tractors with ≥100 horsepower by county	10.1 (8.5)	7.8	5.1	11.0
Percent crop and pasture land by county	33.6 (20.3)	33.7	14.3	51.1
Stream miles per square mile of crop and pasture land by county	2.2 (1.9)	1.5	1.0	2.3
Median size of farms in acres by county	81.1 (21.7)	80.0	63.0	99.0

Univariate associations of the variables of interest with the outcome are presented in Table 4. Table 4 shows that all variables met the p-value cut-off of 0.20, and thus were all further assessed in the multiple regression model. The average slope of pasture land (r = 0.30) and the percent of total tractors with less than 40 horsepower (r = 0.34) by county had the highest significant correlation with the outcome.

**Table 4:** Pearson's correlation coefficients between tractor overturns and independent variables.

*n = 119 counties*			
Dependent Variable	Independent Variables	Correlation Coefficient	p-Value
Tractor overturns per 100,000 tractors	Average percent slope of pasture land by county	0.30	0.0008
	Percent of total tractors with <40 horsepower by county	0.34	0.0001
	Percent of total tractors with ≥100 horsepower by county	−0.18	0.0553
	Percent crop and pasture land by county	−0.22	0.0164
	Stream miles per square mile of crop and pasture land by county	0.19	0.0427
	Median size of farms in acres by county	0.13	0.1663

After including all six variables in the linear regression model and then removing those non-significant variables one at a time, the final model included two significant variables (at p<0.001). The final multiple linear regression model (Table 5) shows that average slope of pasture land (b-estimate = 0.85, 95% CI 0.41, 1.3) and the percent of total tractors with less than 40 horsepower (b-estimate = 0.23, 95% CI 0.12, 0.34) were statistically significantly associated with the outcome of tractor overturns per 100,000 tractors across Kentucky counties. That is, for every 1% increase in average percent slope of pasture land by county, the tractor overturn rate increases by an average of 0.85 per 100,000 tractors (or by 4.25 per 100,000 for every 5% increase in slope). Similarly, for every 1% increase in the percent of total tractors with less than 40 horsepower by county, the tractor overturn rate increases by 0.23 per 100,000 tractors (or by 2.3 per 100,000 for every 10% increase in the total tractors with less than 40 horsepower). No confounding was detected, as removal of variables one at a time did not affect the final two variables in the model.

**Table 5:** Final multiple linear regression model.

*Dependent variable = Tractor overturns per 100,000 tractors*							
*n = 119 counties*							
Variables	Parameter Estimate	Standard Error	t-Value	p-Value	Lower 95% CI	Upper 95% CI	Variance Inflation
Average percent slope of pasture land by county (for every 1% increase)	0.85	0.23	3.8	0.0002	0.41	1.3	1.00
Percent of total tractors with <40 horsepower by county (for every 1% increase)	0.23	0.05	4.3	<0.0001	0.12	0.34	1.00
	R-Square	0.22					
	Adjusted R-Square	0.20					

The final model was highly significant (p<0.0001), with goodness of fit test *R*-square of 0.22 and adjusted *R*-square of 0.20. The model included two un-highly correlated (r = −0.02) variables that did not significantly interact. The global Moran's I value for the outcome alone was significant (Moran's I = 0.26, p<0.01), indicating spatial autocorrelation and a rejection of the null hypothesis of complete spatial randomness of the outcome (Figure 3). However, the residuals of the final model were tested for spatial autocorrelation (i.e., spatial independence of observations), and revealed a non-significant Moran's I value (Moran's I = −0.08, p-value = 0.22), meaning that there was no residual spatial autocorrelation. Thus, the final model can be interpreted as explaining 20% of the spatial patterns observed in the outcome.

## Discussion

As expected, the spatial cluster analysis revealed hotspots (i.e., nonrandom spatial distribution) of tractor overturns, with the greatest risk for overturns in four northern and ten eastern, specifically Appalachian, Kentucky, counties. The regression analysis found that the variation in the distribution of tractor overturns by county can be explained by average slope of pasture land and the percent of total tractors with less than 40 horsepower. These two key ecological findings are consistent with the individual-level observations that lower horsepower tractors have a record of a higher frequency of overturning as do areas with higher slopes [4], [5]. When the results of the different methodologies (SEB smoothing [Figure 2], cluster detection analysis [Figure 3], cartographic visualization of independent variables [Figure 4], and regression modeling [Table 5]) are concomitantly examined, they bolster the evidence of the existence of a high-risk cluster in northern and eastern Kentucky explained by a greater average slope of pasture land and a greater proportion of smaller tractors in those areas.

The aim of this ecological study was to test for high-risk clusters and assess the impact of physiographic and tractor characteristics on overturns, while excluding other individual-level risk factors. We did not aim to test the full range of overturn risk factors that are potentially associated with tractor overturns in the epidemiologic triad (i.e., agent, host, and environment). For instance, we did not test whether factors such as fatigue or age of operator, or environmental factors, such as average rainfall, increased the risk of overturns. While our study focused only on a few risk factors, thus a narrow dimension of the epidemiologic triad, we still found two variables significantly associated with overturns at the county level, explaining 20% of the variation in overturns rates.

To our knowledge this is the first study to investigate high-risk clusters of tractor overturns in conjunction with linear regression modeling at the county level. This study allows for a better understanding of where to target resources and prevention efforts at the county level to reduce tractor overturns in high-risk areas [40], [41]. Targeted interventions in high-risk regions are most appropriate in this context because overturns have been shown to be non-randomly distributed with greatest risk in two key areas, and also because overturns are relatively rare events across many Kentucky counties. Conversely, targeting whole populations across all of Kentucky would mean that resources would be directed at large low-risk populations.

ROPS are a proven intervention in reducing the injury severity of tractor overturns [42], [43]. Though fitting every tractor in Kentucky with ROPS would be ideal, it is not possible given economical constraints. Alternatively, focusing on those areas at greatest risk for overturns may be a more achievable intervention, and also prove more cost effective as the greatest burden of overturns is only spread across 14 counties. This targeted approach may also be more attractive than a population-based approach because both the average slope of pasture land and the proportion of tractors with less than 40 horsepower are relatively immutable. Given these non-modifiable ecological risk factors, it is logical to conclude that the greatest overturn risk will likely persist in time within the same regions, assuming all other factors remain constant. This is further evidence that targeted interventions may be more appropriate than population-based ones.

The next step should be a more targeted ROPS intervention in the areas of greatest risk. This formulation closely follows the National Institute for Occupational Safety and Health's (NIOSH) perspective on tractor related hazards as elucidated by Myers (1998) [44]. Further, as Cole (2007) describes, the steps for a successful ROPS campaign includes “…(1) identifying and targeting farmers most at risk of overturn death and injury, (2) providing these farmers with information about ROPS, and (3) assisting them in acquiring ROPS-protected tractors” [45]. Cole (2007) also suggested that tractors within the six states at most risk for overturn fatalities may be older and without ROPS due to financial constraints. Though our dataset did not contain information on tractor age, it is reasonable to assume that farms within the eastern Kentucky cluster have limited resources given the economic conditions of the region [46], and thus more likely to have older tractors without ROPS. In advancing the National Agricultural Tractor Safety Initiative, we recommend targeted ROPS interventions in the two clusters of greatest risk.

### Strengths, limitations, and future research

Strengths include the diversity of methods incorporated in the study that all coalesce to offer supportive evidence of the presence of high-risk clusters in northern and eastern Kentucky; the use of the spatial scan statistic which eliminates preselection bias and tests for complete spatial randomness, rather than assuming independence of observations as other epidemiological methods; and the first county-level assessment of overturn risk in one of the six states with the highest overturn fatality rates in the nation [9].

This study incorporated SEB smoothing to reduce the problem of instability of variances and population size heterogeneity across counties by adjusting rates based on spatial contiguous neighbors, thereby creating a smoothed visualization of rates. Although SEB smoothing allows for a clearer visualization of spatial patterns, it can also introduce spatial artifacts [47], [48] and thus, is recommended strictly for visualization rather than statistical analyses [49]. This technique is also limited by an edge effect in that counties with fewer contiguous neighbors (i.e., on the edge of the study area) are essentially smoothed less than interior counties that are influenced by all surrounding counties [50]. This study's wide time frame (1960–2002) also makes it vulnerable to recall bias, in that farmers are more likely to recall latter than earlier overturns. Moreover, the KY T/O survey was telephone-based, increasing the risk of selection bias [51]. Also, the denominator (tractor population) used to calculate the overturn rate per county was abstracted from the 2007 Census of Ag, which better reflects tractor population figures in the latter part of the study period than the earlier part. Finally, the regression analysis did not account for all possible confounders associated with tractor overturns.

In guiding future tractor overturn research, it is recommended that individual-level data be collected to improve the understanding of the characteristics of tractor operators within clusters. As risk may not be uniformly distributed within counties, surveillance efforts should seek to collect point data (i.e., latitude, longitude) of the exact location of overturns, so similar analysis can be performed at the census-tract level within the two identified clusters in Kentucky. This type of small-area spatial cluster analysis would allow a finer visualization of those census-tracts with the greatest overturn risk.

### Conclusions

This study found high-risk clusters of tractor overturns in northern and eastern Kentucky and demonstrated the usefulness of the combination and complementary nature of spatial statistics and traditional regression in identifying areas at highest risk for overturns. Our results can guide intervention efforts at the county level in Kentucky to reduce overturns and overturn injury severity in those areas at greatest risk.
